# The reconvictions of mentally disordered offenders—how, when, and where?

**DOI:** 10.1186/s12888-022-03912-4

**Published:** 2022-04-13

**Authors:** Ebba Noland, Mattias Strandh, Fia Klötz Logan

**Affiliations:** 1 Department of Social Work, Umeå University, 901 87 Umeå, Sweden; 2 Sundsvall Forensic Psychiatric Centre, Region Västernorrland, Box 880, 851 24 Sundsvall, Sweden

**Keywords:** Forensic psychiatry, Mentally disordered offenders, Criminal recidivism, Reconviction

## Abstract

**Background:**

Little is known about the recidivism of mentally disordered offenders after discharge from forensic psychiatric services. This is problematic because such knowledge could (i) help professionals who encounter this group to better plan interventions to prevent recidivism, (ii) clarify the rates of recidivism post-discharge from forensic psychiatric care and (iii) further develop instruments for specific risk assessment. The aim of this study was to investigate the new crimes of mentally disordered offenders who had been reconvicted after discharge from forensic psychiatric care.

**Methods:**

Included in this study were all individuals (*n* = 1142) who had been discharged from forensic psychiatric care in Sweden during 2009–2018, were included in the Swedish National Forensic Psychiatric Register, and had been reconvicted in a criminal court within the follow-up period of 2009–2018 (*n* = 157, 14% of the population). The follow-up times of the discharged patients within the period varied from 4 to 3644 days, (*m* = 1697, *Md* = 1685). Retrospective registry data along with coded data from criminal court judgments (*n* = 210) were used to create a database. Kaplan–Meier survival analysis and descriptive statistical analysis was performed.

**Results:**

75% of included individuals were reconvicted for at least one violent crime, but only 9 individuals were reconvicted for a serious violent crime, which can be compared to the 44 individuals with serious violent index crimes. The most common crime was “Other violent”. The most common sentence was probation. The offender’s most common relationship to the victim was having no known relationship, followed by the victim being a person of authority. The most common circumstance of the crime leading to the reconviction was that it occurred without apparent provocation; other common circumstances were related to the exercise of public authority. The most common crime scene was a public place.

**Conclusions:**

Even though the reconvictions of this group included many violent crimes, there were very few serious violent crimes. The findings that the victims of the crimes of mentally disordered offenders are most commonly either unknown to the perpetrator or persons of authority, and that the crimes are often perpetrated without apparent provocation or reason, are important information for all professionals who encounter this group and should be taken into consideration to assess risk more accurately.

**Supplementary Information:**

The online version contains supplementary material available at 10.1186/s12888-022-03912-4.

## Background

Mental disorders, and therefore also mentally disordered offenders (MDOs), exist in all countries [1]. The Swedish Penal Code, however, differs from most others in the world since it states that mental disorders do not absolve a defendant from criminal responsibility. If the defendant who is convicted of a crime is also found to have a severe mental disorder and a need for psychiatric care at the time of the trial, they will be sentenced to forensic psychiatric care [2]. The mission of forensic psychiatric care is to improve the patient’s mental health and prevent future recidivism [3]. Swedish legislation states that for a forensic psychiatric patient to be discharged from forensic psychiatric services, both the risk of reoffending with a serious crime due to mental disorder and the patient’s mental state and personal condition must be considered [4]. Data on post-discharge recidivism is thus essential for evaluating forensic psychiatric services and their discharge processes. However, research on the nature of recidivism among former forensic psychiatric patients is scarce.

Previous studies have found that the factors most strongly associated with an increased likelihood of recidivism among MDOs include number of previous convictions, younger age at first conviction, and substance abuse [5–10], with personality disorder linked to the highest risk of violent offending [11]. Although criminal history and actuarial variables were the best predictors for reoffending [7], dynamic risk factors have also been showed to be good predictors of desistance from future violence [12]. Furthermore, situational factors including having a trustee/limited guardian and living mainly in supported accommodation were associated with a reduced likelihood of recidivism [13]. When planning for the future, all risk factors in a person’s life need to be taken into consideration [14]. 

An earlier report found that 11% of forensic psychiatric patients had been reconvicted within one year of discharge from forensic psychiatric care, and around 30% had been reconvicted after five years [15]. For comparative purposes, Fazel et al. [16] found that with a mean follow-up time of 3.2 years, 59% of offenders had reoffended after being released from prison. The rate of recidivism among MDOs is thus relatively low when compared to offenders in general [17] even though MDOs have more general risk factors than other offenders [18], suggesting that research on the recidivism of offenders in general may not be generalizable to the recidivism of MDOs. Specific studies on recidivism among MDOs following discharge from forensic psychiatric care are therefore needed.

Today, there is limited information on the nature of the recidivism perpetrated by MDOs. The Swedish National Forensic Psychiatric Register (SNFPR) has reported that of the discharged MDOs who were reconvicted within a year, 26% were reconvicted of violent crime, 29% for crime against property, and 45% for other crime. The SNFPR also reported violent recidivism to be the most common form of recidivism, and that reconviction for the same type of crime as the index crime (the crime for which the person was convicted to forensic psychiatric care) was more common for violent crime than for other types of crime [15]. Previous research has also shown that MDOs with severe violent index offences were less likely to reoffend than those with less serious index offences [19], and that repetition of serious violence is rare [9].

Alm et al. identified gender differences in the patterns of reoffending of MDOs after discharge from forensic psychiatric services [20], but it was noted that its findings should be interpreted with caution due to the study’s small sample size (only 13 women and 23 men in the sample reoffended). Another study on the subject included only 24 patients with reconvictions [9]; small sample sizes is a common problem in forensic psychiatric research. This is largely because the relatively small overall number of forensic psychiatric patients and the group’s low rate of recidivism means that studies on recidivism of MDOs often have low statistical power.

The lack of research on the subject means that we do not know sufficiently how, where, and against who MDOs who reoffend commit their post discharge crimes. This is problematic because such knowledge is needed to plan appropriate interventions to prevent recidivism, evaluate the rates of reoffending post discharge from forensic psychiatric care, and to further develop instruments for specific risk assessment. Having more knowledge on this subject is also important because it would help professionals such as the police, social workers, and mental health professionals who encounter this group to better plan appropriate interventions and prevent future recidivism. The aim of this study was to investigate the new crimes of MDOs who had been reconvicted after discharge from forensic psychiatric services, investigating the characteristics of the reconvictions and the type of reconviction as compared to the index crime of the individual.

## Methods

### Study design

This study examined both retrospective registry data and criminal court judgments. The registry data was primarily collected from the SNFPR. A database was constructed by adding data on criminal sentencing post discharge from the National Council of Crime Prevention (NCCP), information on year of birth from Statistics Sweden, and coded data from district court criminal judgments. Data from the NCCP was used to identify patients who had been reconvicted after discharge from forensic psychiatric care within the follow-up period. All the new court judgments concerning these individuals (*n* = 210) were requested separately from Sweden’s district courts (of which there are 48). The court judgments were read separately by one of the authors (EN) and coded.

### Study sample

Included were all individuals discharged from forensic psychiatric care in Sweden during 2009–2018 who were included in the SNFPR and had been reconvicted in a criminal court during the follow-up period of 2009–2018. At the time of the data collection, the SNFPR included 86% of all forensic psychiatric patients in Sweden [17]. The remaining 14% of patient not included in the register had either not been asked to participate or declined the request.

The group of all patients discharged within the period consisted of 1142 individuals, 935 (82%) men and 207 (18%) women. The follow-up times of the discharged patients within the period varied from 4 to 3644 days, (*m* = 1697, *Md* = 1685). Of these, 157 individuals were reconvicted and therefore included in this study, of which 139 (89%) were men and 18 (12%) were women. For further descriptive statistics, see Table 1. As exact date of the criminal relapse was inconsistently reported in the court judgments, the date of the sentencing was used.

**Table 1:** Data on reconvictions post discharge from forensic psychiatric care

	All (*n* = 157)
	*M (Md)*
Time from discharge to new conviction (months)	34 (27)
Number of crimes/person during follow-up	3.8 (2)
Number of victims in court judgment	2.4 (1)
Number of convictions during follow-up	1.4 (1)
	***N (%)***
Reconvicted for any violent crime	118 (75.2)
Reconvicted for any serious violent crime	9 (5.7)

### Coding of court judgments

For all variables, only the information described in the court judgment was coded; any variable not explicitly included in the court judgment was coded as “missing”.

#### Crime category

The crimes were categorized as violent or non-violent, using the same definition as previous Swedish research [5]. The following crimes were classified as violent: homicide, manslaughter, assault and battery, arson, unlawful threats, violation of integrity, unlawful coercion, molestation, violence against an officer, robbery and sexual offenses including sexual molestation. The categories of violent and non-violent crime were then divided into subcategories. The subcategories of violent crime were lethal violence (including attempted lethal violence), sexual violence, arson, violence against an officer and other violent crime. The subcategories of non-violent crime were theft, drugs/alcohol, traffic, vandalism, and other non-violent.

#### Serious violent crime

Serious violent crimes were defined as homicide, manslaughter, aggravated assault, sexual offences, and arson, including attempts to commit these crimes. This definition has previously been used in Swedish research [9] on the recidivism of MDOs.

#### Sentence

The most severe sentence in each court judgment was registered. Sentences depriving the defendant of their liberty were considered the most severe, followed by conditional sentences and probation. Least severe was having to pay fines.

#### Relationship to victim

Individual court judgments may address crimes against multiple victims. This variable measures the number of judgements in which the defendant had a given relationship to any of the victims, without counting the number of victims of each category. The categories used were: partner/ex-partner, family member, acquaintance, authority figure, other, no known relationship, and no victim. Coding for this variable was performed based on the text of the court judgment. Crimes with legal entities as victims were coded as “no known relationship”; in the court judgments where “no known relationship” was registered, 26% of the victims were legal entities.

#### Circumstance of crime

The circumstance of the crime was defined as the situation that preceded the crime or explains in what situation the crime was committed. Because an individual court judgment may address several crimes, each court judgment could include several circumstances. This variable measures the number of court judgments in which a given circumstance could be identified. The circumstances considered were: traffic, narcotics, weapons crime, exercise of public authority, related to victim’s work (not public authority), burglary, theft/robbery not in victim’s home, economic crime, social gathering, stalking, intimate partner violence, sexual crime, motivated by previous injustice, correction/refusal of something, no apparent provocation, and other.

#### Crime scene

Since an individual court judgment may address multiple crimes with different crime scenes, each court judgment could have several different crime scenes. This variable measures the number of court judgments in which the crime was committed in a given type of location. The categories used were: public place, victim’s home/vehicle, defendant’s home/vehicle, third party’s home/vehicle, care institution/administrative authority, from a distance, and other (which included semi-public locations such as privately-owned shops, schools, etc.). This was coded based on the text of the court judgment; if the court judgment provided no information on the crime scene, the variable was coded as “missing”.

### Data analysis

Kaplan–Meier survival curves for the time to reconviction were plotted, comparing men to women and individuals with a violent index crime to individuals with a non-violent index crime. The overall difference between survival curves was calculated using the log-rank test. Descriptive statistics including frequencies, means, and medians for the new convictions were calculated.

## Results

### Survival

**Fig. 1:**
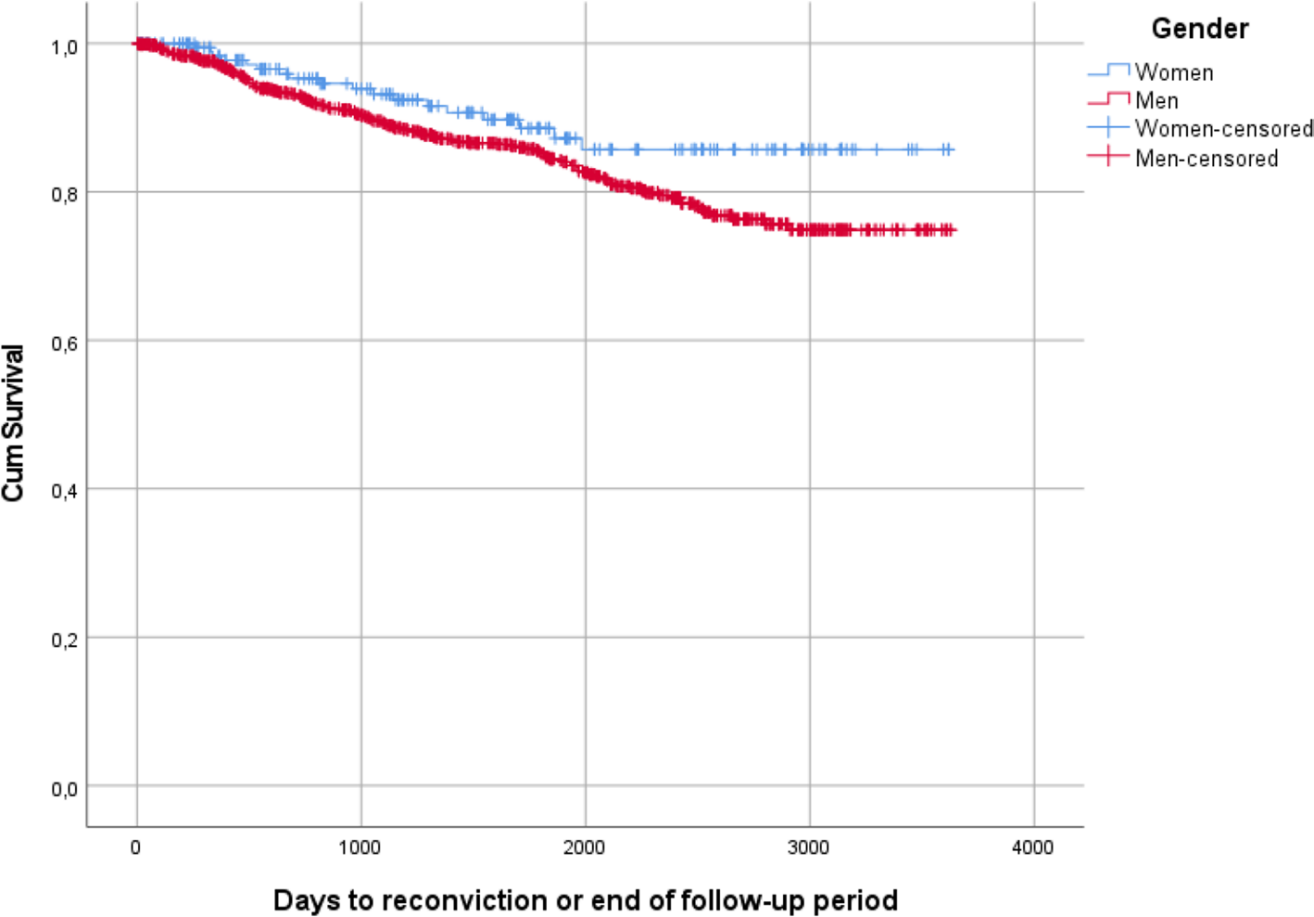
Kaplan–Meier estimates of time to reconviction for men and women

Of the whole group of MDOs discharged from forensic psychiatric care (*n* = 1142), most individuals (86.3%) were not reconvicted within the follow-up period. Figure 1 shows the Kaplan–Meier curves for the rate of recidivism in the whole group, according to sex. Of the women (*n* = 207), 8.7% were reconvicted and of the men (*n* = 935) 14.9% were reconvicted. The differences were not statistically significant (*p* = 0.06), however, as this was the entire population, significance levels are not entirely meaningful. The curves show that the risk for reconviction for both groups was evenly distributed over time although greater during the first years post-discharge.

### Background characteristics

The group consisted of 18 women (11.5%) and 139 (88.5%) men. Their mean length of stay in forensic psychiatric services was 42.6 months, i.e. about 3.5 years (*Md* = 32.4, SD = 38.4), and they were on average 38.03 years old (*Md* = 36, SD = 11.3) at the time of discharge. The follow-up time ranged from 75–3644 days (*M* = 2209.8, *Md* = 2433). For 67.5% of them, the forensic psychiatric care had been combined with special court supervision (SCS). The index crime was most commonly a violent crime (83.4%), with “other violent” (including assault, violent threats and robbery) being the most common (61.1%). The index crime was a serious crime for 44 individuals (28%). For further descriptive background statistics, see Additional file 1.

### The new convictions

In total, 157 MDOs were reconvicted within the follow-up period; see Table 1 for descriptive statistics about the reconvictions and Table 2 for information on the crimes giving rise to the reconvictions. There were 210 new court judgments relating to these individuals; while most (72.6%) were convicted only once during the follow-up period, 16.6% had been convicted twice and 7% had been convicted 3–7 times. No-one had been convicted more than 7 times within the follow-up period. Individual court judgments may address multiple crimes; in total, the court judgments addressed 573 crimes. On average, each MDO was convicted of 3.8 crimes (*Md* = 2). Most of the group, 118 individuals (75.2%), had committed at least one violent crime. However, only nine MDOs (7.6% of those who had committed a new violent crime, 5.7% of all who were reconvicted) were reconvicted for a serious violent crime.

**Table 2:** Types of crime leading to reconvictions post discharge from forensic psychiatric care

	All (*n* = 573) *n* (%)
Violent crimes	
Lethal violence	7 (1.2)
Sexual	9 (1.6)
Arson	4 (.7)
Violence against an officer	60 (10.5)
Other violent	184 (32.1)
Non-violent crimes	
Theft	84 (14.7)
Drugs/alcohol	60 (10.5)
Traffic	51 (8.9)
Vandalism	28 (4.9)
Other non-violent	86 (15.0)

### Type of crime

Table 2 shows that the most common crime was “Other violent” (32.1% of all crimes), which included assault, threats of violence, and robbery. This was followed by “Other non-violent” (15% of all crimes), which included crimes against public activity, illegal carrying/possession of a weapon or knife, and supplementary penal provisions outside the Penal code. Also common were theft (14.7% of all crimes), violence against an officer (10.5% of all crimes), and drug/alcohol-related offences (10.5% of all crimes).

### Sentence

The sentences assigned at the reconvictions are shown in Table 3. The most common sentences were probation (35%), forensic psychiatric care (30%) and prison (27.7%). For court judgments pertaining to at least one violent crime, the most common sentences were forensic psychiatric care (37.6%), probation (30.9%), and prison (24.8%). For those who were sentenced to prison, the average length of the prison sentence was 11.3 months (*Md* = 6 months).

**Table 3:** The reconvictions post discharge from forensic psychiatric care^a^

	All (*n* = 210) *n* (%)	Violent crime (*n* = 149)^b^ *n* (%)
Sentence		
Forensic psychiatric care	65 (30.0)	56 (37.6)
Prison	60 (27.7)	37 (24.8)
Probation	76 (35.0)	46 (30.9)
Conditional sentence	16 (7.4)	10 (6.7)
Relationship to victim		
Partner/ex-partner	21 (11.5)	21 (14.1)
Family member	20 (11.5)	20 (13.4)
Acquaintance	41 (23.5)	38 (25.5)
Authority figure	59 (31.2)	56 (37.6)
Other	2 (1.3)	1 (0.7)
No known relationship	105 (45.9)	72 (48.3)
No victim	34 (17.2)	2 (1.3)
Circumstance of crime		
Traffic	17 (8.3)	3 (2)
Narcotics	23 (12.7)	5 (3.4)
Weapon’s crime	4 (2.5)	1 (0.7)
Exercise of public authority	49 (27.4)	47 (31.5)
Related to victim’s work, not public authority	9 (5.7)	8 (5.4)
Burglary	20 (10.8)	7 (4.7)
Theft/robbery, not in victim’s home	47 (24.2)	19 (12.8)
Economic crime	5 (3.2)	0 (0)
Social gathering	12 (7.0)	12 (8.1)
Stalking	6 (3.8)	6 (4)
Intimate partner violence	18 (10.2)	18 (12.1)
Sexual crime	7 (3.8)	7 (4.7)
Previous injustice	17 (9.6)	16 (10.7)
Correction/refusion of something	10 (6.4)	10 (6.7)
No apparent provocation	62 (33.8)	58 (38.9)
Other	9 (4.3)	7 (4.7)
Crime scene		
Public place	90 (42.3)	64 (43)
Victim’s home/vehicle	50 (23.8)	38 (25.5)
Defendant’s home/vehicle	22 (10.4)	20 (13.4)
Third party’s home/vehicle	10 (4.8)	9 (6)
Care institution/administrative authority	29 (13.8)	26 (17.4)
Other (e.g. Privately-owned shops)	68 (32.4)	42 (28.2)
From a distance	27 (12.9)	18 (12.1)

^a^values shown in the table are the number of court judgments including the indicated sentence and (in parentheses), this number as a percentage of all court judgments included in the study. Note that one court judgment may address multiple crimes

^b^values shown in the table are the number of court judgments pertaining to violent crime including the indicated sentence and (in parentheses), this number as a percentage of all court judgments included in the study

### Victims

In total, 376 victims were registered in all court judgments. Of these 157 (42%) were male, 142 (38%) were female, and 76 (20%) were legal entities. The number of victims in the court judgments ranged from 0 to 18 (*M* = 2.4*, Md* = 1). For court judgments addressing at least one violent crime, there were in total 305 victims. Of these 139 (46%) were male, 124 (41%) were female, and 42 (14%) were legal entities.

The most common relationship to the victim in all court judgments was no known relationship (45.9%), followed by the victim being a person of authority (31.2%), an acquaintance (23.5%), and the crime being victimless (17.2%). It should be noted that for the sentences where “no known relationship” was registered, 50 of the 190 victims (26%) were legal entities.

In court judgments addressing violent crimes, no known relationship was most common (48.3%), followed by the victim being an authority Fig. (37.6%) and the victim being an acquaintance (25.5%). For further information on the relationships between perpetrators and victims, see Table 3.

### Circumstance of crime

The most common circumstance of crime across all court judgments was no apparent provocation (33.8%), followed by exercise of public authority (27.4%), and theft or robbery occurring in a location other than the victim’s living accommodation (24.2%).

For court judgments addressing violent crime(s), the most common circumstance was no apparent provocation (38.9%), followed by exercise of public authority (31.5%) and theft or robbery occurring in a location other than the victim’s living accommodation (12.8%). For more detailed information, see Table 3.

### Crime scene

The most common crime scene in all court judgments was a public place (42.3%). This was followed by “other” (32.4%), which included semi-public locations such as privately-owned shops. Other common crime scenes were the victim’s home/vehicle (23.8%), an institution/administrative authority (13.8% of court judgments), and from a distance (12.9%). For court judgments containing at least one violent crime, the most common crime scene was a public place (43%), “other” (28.2%) and victim’s home or vehicle (25.5%). Other common crime scenes were an institution/administrative authority (17.4%) and the defendant’s home or vehicle (13.4%), see Table 3.

### The index crime and the reconviction

**Fig. 2:**
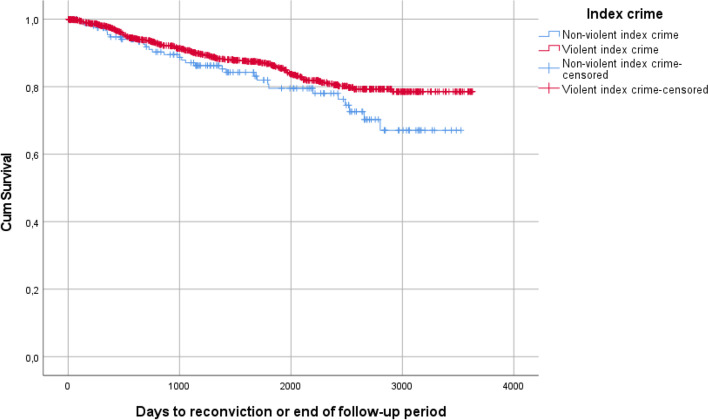
Kaplan–Meier estimates of time to reconviction for individuals with violent and non-violent index crimes

Of all MDOs discharged from forensic psychiatric care (*n* = 1142), 161 (14.1%) had been convicted for a non-violent index crime and 981 (85.9%) had been convicted for a violent index crime. Figure 2 shows the Kaplan–Meier curves according to violent and non-violent index crimes. Of those with non-violent index crimes (*n* = 161), 19.3% were reconvicted and of those with violent index crimes (n = 981) 12.8% were reconvicted. The differences were not statistically significant (*p* = 0.09). however, as this was the entire population, significance levels are not entirely meaningful. The curves show that the risk for reconviction for both groups was greater during the first years post-discharge.

Table 4 shows how many individuals with violent/non-violent index crimes were reconvicted for violent/non-violent crimes. In total, 26 individuals had been convicted for a non-violent index crime. Twenty (76.9%) of these people were subsequently reconvicted for a non-violent crime, 15 (57.7%) were convicted for a non-serious violent crime, and none were convicted for a serious violent crime. Eighty-seven individuals had been convicted for a non-serious violent index crime, of whom 61 (70.1%) were later reconvicted for a non-violent crime, 69 (79.3%) were reconvicted for a non-serious violent crime, and 5 (5.7%) were reconvicted for a serious violent crime. Forty-four individuals had been convicted for a serious violent index crime, of whom 29 (66%) were later reconvicted for a non-violent crime, 34 (77.2%) were reconvicted for a non-serious violent crime, and 4 (9.1%) were reconvicted for a serious violent crime.

**Table 4:** Number of mentally disordered offenders reconvicted for violent/non-violent crime

	Reconviction	Reconviction
	Non-violent, (%)	Violent, any (%), **of which serious (%)**
Index crime	20 (77)	15 (58), **0 (0)**
Non-violent (*n* = 26)		
Index crime	61 (70)	69 (79), **5 (6)**
Violent, not serious (*n* = 87)		
Index crime	29 (66)	34 (77), **4 (9)**
Serious violent (*n* = 44)		
Total (*n* = 157)	110 (70)	118 (75), **9 (6)**

## Discussion

The aim of this study was to investigate the post-discharge crimes of MDOs. Kaplan-Meier curves showed a trend where men were more often reconvicted than women, as was individuals with non-violent index crime compared to individuals with violent index crimes. These results did not reach statistical significance, but as this population is nearly the whole population, significance levels are not entirely meaningful.

The group of MDOs who are reconvicted after discharge from forensic psychiatric services consists mainly of men. Typically, they are in their late thirties at the time of discharge, have a violent index crime (even though the individuals with non-violent index crimes were more often reconvicted, there was a larger number of individuals with a violent index crime in the reconvicted group) and a history of substance abuse, and have been diagnosed with some form of psychosis. The full group of patients discharged from forensic psychiatric care during the studied period shares these characteristics, although the reconvicted individuals as a group were slightly younger at the time of discharge [13]. It should also be noted that most of the MDOs discharged during this period (86%) were not reconvicted. As previous research using longer follow-up periods have shown higher rates of recidivism [5, 21], it is possible that recidivism for MDOs generally occurs later than what this study design is able to show. The Kaplan–Meier survival analyses of this study, however, suggest that this is not the case.

Most of the reconvicted individuals had one new conviction during the follow-up period, but it was common for individual court judgments to address multiple crimes. Most of the group had been reconvicted for at least one violent crime and at least one non-violent crime. However, the proportion of the individuals convicted for serious violent crimes was significantly lower for the reconvictions (6%) than for the index crimes (28%), which is in line with previous research [9]. This indicates that even when this group reoffended, their criminality was generally less severe than before receiving forensic psychiatric care. The conclusion that recidivism was less serious overall than the index crimes is supported by the fact that the most common sentence in all judgments was probation (see Table 4), which is a milder sentence than prison or forensic psychiatric care. The high rate of probation may also reflect the high frequency of substance abuse in the group, because Swedish law allows probationary sentences to be combined with compulsory treatment for substance-related disorders [22]. As so few (*n* = 9) were reconvicted for serious violent crimes, no statistical analyses were performed to compare this group to the other individuals who had reoffended, but this would be an interesting topic for future research.

If only court judgments addressing at least one violent crime are considered (149 of the 210 judgments), most of the above remains true. The relative frequencies of the different relationships between perpetrators and victims were similar, and the different circumstances (aside from those relating specifically to thefts) were roughly as common as in the full set of court judgments. However, among court judgments for violent crimes, sentences to forensic psychiatric care were more common, fewer victims were legal entities, and the crime scene was more often a care institution or administrative authority and more rarely categorized as “other” (which often meant semi-public places such as privately-owned shops).

### No apparent provocation and stranger victims

The most commonly observed circumstance of crime was “No apparent provocation”, and the most common relationship between the MDO and their victim was having no known relationship, followed by the victim being an authority figure, for example a police officer, social worker or health personnel. Even though “no known relationship” includes crimes with legal entities (such as organizations or companies) as the victim, most crimes in this group were committed against an actual person. This is in line with previous research showing that compared to offenders without mental illness, offenders with schizophrenia were more likely to offend in open places and to target strangers [23]. These facts support the stereotypical image of crimes perpetrated by mentally disordered individuals that is commonly presented in the media and was summarized well by FE Markowitz [24], who described how the high publicity surrounding certain violent events has generated misunderstandings concerning the risk of violence and a perception of mental illness that overemphasizes violence. However, the real crimes committed by MDOs after discharge from forensic psychiatric care are generally less serious than the stereotype suggests. This is important because it has also been shown that concerning offenders in general, imprisonment does not reduce recidivism [25] and that an individual’s number of convictions is associated with an increase in seriousness of crime [26]. It has also been reported that while the rate of stranger perpetrators differs between types of crime, only a third of all violent crime is perpetrated by strangers [27]. The higher rate of stranger perpetrators and the generally less serious recidivism shown in this study strengthens the assumption that MDOs are a specific group requiring further study.

The high frequency of crimes against persons of authority also highlights situations of increased risk that may need to be considered by forensic psychiatric services before discharging a patient. It is also something that professionals of different kinds should be aware of when encountering MDOs, along with the fact that reconvicted MDOs with a violent index crime are more likely to be reconvicted for a violent crime than those with a non-violent index crime. This is supported by previous research showing that a history of violence is a very important risk factor for future violence [14]. It may be assumed that providing training specifically in dealing with MDOs (see for example M Ahern [28]) and pre-discharge training of MDOs to prepare for such situations might reduce the number of crimes against this group.

Another notable finding is the relatively high frequency of crimes involving some sort of (perceived) previous injustice or refusal. This could be an issue for forensic psychiatric services to address before discharge, which could be done both by investigating whether there is someone the patient holds a grudge towards and by focusing interventions on how the patient handles perceived injustices and refusals.

### The assessments leading to discharge

It should be noted that when evaluating a patient for discharge from forensic psychiatric care combined with SCS, one factor that must be considered by the court or chief medical officer is the risk of reoffending with a *serious* crime [4]. The fact that much of the recidivism of MDOs seems to involve minor offenses and the rarity of serious crimes suggests that this aspect of the system works reasonably well. The effectiveness of the current system (possibly including both the forensic psychiatric care that is provided and the procedure for discharging patients) is also supported by the fact that the overall recidivism rate of MDOs is low when compared to that of former prisoners overall [15, 16]. However, the reasons for this and the influence of forensic psychiatric care on the rate of recidivism remain unclear, particularly since previous studies on offenders referred to pre-trial psychiatric investigations have revealed no significant differences between individuals sentenced to forensic psychiatric treatment, prison, or noncustodial sanctions [5].

### New forensic psychiatric care – a sign of not taking prescribed medication?

The second most common sentence among the studied MDOs was committal to forensic psychiatric care, indicating that these individuals were once again assessed as needing inpatient forensic psychiatric care. This is not entirely surprising since MDOs discharged from forensic psychiatric services have previously been acknowledged to have high levels of continued psychiatric morbidity [29] and hospital readmission [30]. However, it has been shown that the crimes of MDOs were rarely directly motivated by symptoms [31]. Previous research on patients with schizophrenia also implies that offending behaviors reflects a range of factors from before, during and after active illness [32], which may be the case for MDOs in general as well. Further investigation on whether these patients’ mental states had worsened and led to reoffending because they had stopped taking their prescribed medication since discharge would nevertheless be highly interesting, especially since Canadian research has shown that medication non-compliance increased significantly in the year following absolute discharge [33].

### Limitations

A limitation of this study was the fact that Swedish court judgments vary widely in terms of the detail they provide about the circumstances of the crime. This affected the coding process, making it impossible in some cases to determine which circumstances applied or whether the perpetrator and victim knew one-another. As a result, some data were categorized as “missing”. However, the sample size is still considerably larger than those of previous studies on recidivism among MDOs. It should also be noted that this study includes only recidivism after discharge from forensic psychiatric care and not the recidivism that might have happened during the care, even though we know that reconvictions happen during the care as well [34].

## Conclusion

Although the reconvictions of this group included many violent crimes, only a few of these crimes were of a more serious nature. The goal of the forensic psychiatric services is obviously to prevent reoffending entirely, but it seems that at least the reconvictions of MDOs are generally for crimes less serious than the index crimes.

The finding that the victims of the MDOs’ crimes are most often unknown to the perpetrator, and that the crimes are often perpetrated without apparent provocation or reason are important information for all professionals who encounter this group. Furthermore, it was found that persons of authority are at higher risk of becoming victims when exercising their authority. These finding could be of use to further develop instruments for specific risk assessment, as well as improving the planning of security measures.

## Supplementary Information

**Additional file 1.** Characteristics of mentally disordered offenders who were both discharged and reconvicted between 2009 and 2018.

## Data Availability

The data that support the findings of this study are available from SNFPR but restrictions apply to the availability of these data, which were used under license for the current study, and so are not publicly available. Data are however available from the authors upon reasonable request and with permission of SNFPR.

## Abbreviations

MDO: Mentally disordered offender
SCS: Special court supervision
SNFPR: Swedish National Forensic Psychiatric Register
NCCP: National Council of Crime Prevention
