# Supplementary material for: The reconvictions of mentally disordered offenders—how, when, and where?
Source: BMC Psychiatry. 2022 Apr 13;22:264. doi: 10.1186/s12888-022-03912-4 (PMC9008909; doi:10.1186/s12888-022-03912-4)
Supplement: Supplementary file 1 — Additional file 1. Characteristics of mentally disordered offenders who were both discharged and reconvicted between 2009 and 2018. [file 12888_2022_3912_MOESM1_ESM.docx]

**Appendix 1.** Characteristics of mentally disordered offenders who were both discharged and reconvicted between 2009 and 2018.

|  | All (*n*=157) |
| --- | --- |
|  | *M (Md)* |
| Age at discharge | 38.03 (36.00) |
| Length of stay in forensic psychiatric services (months) | 42.60 (32.37) |
| Follow-up time (days) | 2209.81 (2433.00) |
|  | ***N (%)*** |
| Sex |  |
| Men | 139 (88.5) |
| Women | 18 (11.5) |
| Index crime |  |
| Serious violent | 44 (28.0) |
| Non-serious violent | 87 (55.4) |
| Non-violent | 26 (16.6) |
| Special court supervision | 106 (67.5) |
| Diagnosis |  |
| Psychosis, no PD | 77 (49.0) |
| PD, no psychosis | 30 (19.1) |
| Psychosis and PD | 10 (6.4) |
| Neither psychosis nor PD | 40 (25.5) |
| History of substance abuse | 114 (72.6) |
